# Risk factors for cerebral infarction and cerebrovascular stenosis in antiphospholipid antibody-positive patients: A retrospective single-center study with propensity score matching analysis

**DOI:** 10.1097/MD.0000000000039890

**Published:** 2024-09-27

**Authors:** Seung Hyun Ha, Sang-Uk Kim, Joon Huh, Choon-Woong Huh

**Affiliations:** a Department of Neurology, Myong-Ji St. Mary’s Hospital, Seoul, Republic of Korea; b Department of Neurosurgery, Myong-Ji St. Mary’s Hospital, Seoul, Republic of Korea.

**Keywords:** anticardiolipin, antiphospholipid antibodies, antiphospholipid syndrome, cerebrovascular disorders, comparative studies, ischemic stroke

## Abstract

Antiphospholipid syndrome (APS) is an autoimmune disorder characterized by the presence of antiphospholipid antibodies (aPLA), such as anticardiolipin (aCL), anti-β2-glycoprotein I (aβ2GPI), or lupus anticoagulant (LA). Although cerebrovascular events are commonly associated with APS, comprehensive studies on risk factors for cerebral infarction in aPLA-positive patients remain sparse. In this retrospective single-center study, data from 9844 patients tested for aPLA between January 2017 and March 2023 were analyzed. A total of 647 aPLA-positive patients were included, with assessments of various factors including age, gender, hypertension, diabetes, dyslipidemia, smoking history, and cardiac disease. Propensity score matching was employed to create 2 matched groups of 202 patients each, comparing those with and without cerebral infarction. Logistic regression analyses were conducted to identify risk factors for cerebral infarction and progression of cerebrovascular stenosis. The mean age of the study cohort was 65.8 years, with 60% being male. LA was positive in 95.2% of the cases, aCL in 8.8%, and aβ2GPI in 5.3%. High-risk aPLA profiles were identified in 7.1% of the cases. In the cerebral infarction group, both smoking history and aCL positivity were significantly associated with an increased risk (OR = 1.543; 95% CI: 1.020–2.334; *P* = .040 and OR = 3.043; 95% CI: 1.426–6.491; *P* = .040, respectively). Male gender and posterior circulation involvement were significant risk factors for exacerbation of cerebrovascular stenosis (OR = 3.73; 95% CI: 1.16–16.69; *P* = .046 and OR = 5.41; 95% CI: 1.80–16.05; *P* = .002, respectively). Smoking history and aCL positivity are prominent risk factors for cerebral infarction in aPLA-positive patients, while male gender and involvement of the posterior circulation emerge as significant risk factors for the progression of cerebrovascular stenosis. Further comprehensive prospective studies are necessary to deepen understanding of aPLA-related cerebrovascular diseases.

## 1. Introduction

Antiphospholipid syndrome (APS) is an acquired autoimmune disorder characterized by the presence of antiphospholipid antibodies (aPLA) such as IgG/IgM anticardiolipin (aCL), anti-β2-glycoprotein I antibodies (aβ2GPI), or lupus anticoagulant (LA). This syndrome manifests through arterial, venous, or microvascular thrombosis, and/or obstetric morbidity, which includes recurrent early miscarriages, late pregnancy losses, stillbirth, and placental insufficiency. APS is relatively rare, with an estimated incidence of 1 to 2 cases per 100,000 and a prevalence of 40 to 50 per 100,000. Notably, a study by Hwang et al, reported an incidence of 0.75 and a prevalence of 6.2 per 100,000 in Korea, suggesting lower rates in Asian populations.^[1–3]^

Acute ischemic strokes frequently occur in individuals with underlying vascular and metabolic risk factors such as hypertension, dyslipidemia, diabetes mellitus, and smoking. It is significant, however, that 20% of APS patients experience strokes. Additionally, 17% of individuals under the age of 50 who have experienced strokes and 12% of those with transient ischemic attacks (TIA) are associated with aPLA. In instances where the cause of stroke or related symptoms in young adults remains unexplained, identifying conditions such as APS becomes crucial for appropriate medical management.^[4–6]^

Several studies have demonstrated the prothrombotic activation associated with each type of aPLA and the correlation between aPLA positivity and cerebrovascular events along with clinical characteristics.^[7–10]^ Nevertheless, there is a scarcity of detailed research on the specific characteristics of each antibody and the risk factors for cerebral infarction in individuals positive for aPLA. Therefore, our single-center study aims to analyze the risk factors for cerebral infarction in the aPLA-positive patient group through the use of propensity score matching (PSM).

## 2. Materials and methods

### 2.1. Study population and data collection

**Figure 1.**
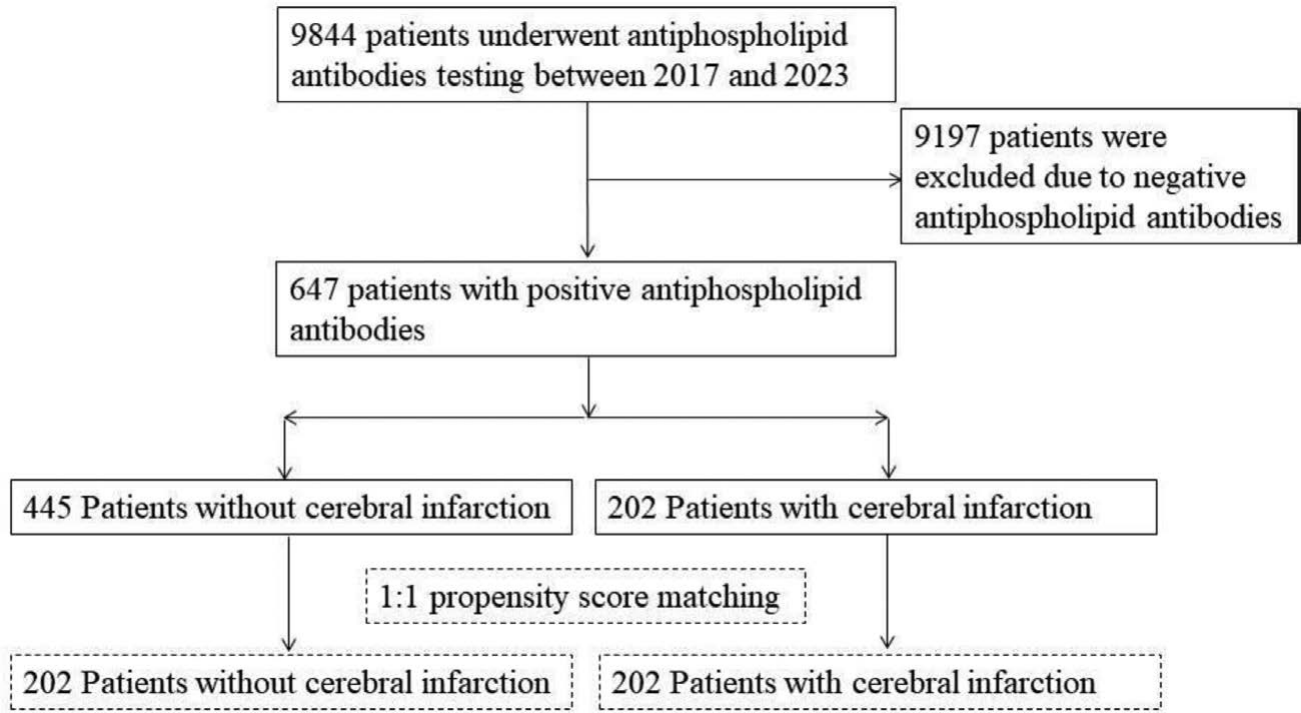
Flow chart of study participants.

This study was conducted retrospectively at a single institution. Between January 2017 and March 2023, data were collected on 9844 patients who underwent aPLA testing for headache, stroke, TIA, and APS diagnosis at our hospital. Of these, 647 patients were included, while 9197 patients were excluded due to all-negative results. Patient-related factors such as age, gender, hypertension, diabetes, dyslipidemia, smoking history, and cardiac disease were assessed through a retrospective review of medical records. Imaging studies were also reviewed to identify cerebral infarction and cerebrovascular lesions. Subsequently, 202 patients with cerebral infarction and 445 patients without were categorized into 2 groups. As depicted in Figure 1, 1:1 PSM was conducted, and subsequent research analysis was performed on 2 matched groups of 202 patients. The Institutional Review Board of the author’s institution approved this study (MSH-2024001).

## 3. aPLA testing and aGAPSS score calculation

Immune serological testing was conducted for the 3 essential elements in the diagnosis of antiphospholipid syndrome, according to the updated Sapporo criteria: LA, aCL, and aβ2GPI.^[11]^ Plasma samples for LA measurement were analyzed using a coagulant machine (STA R MAX, STAGO, Asnières sur Seine, France) and STACLOT DRVV reagent kit (STAGO), adhering to the manufacturer’s guidelines. Measurement of IgG/IgM for aCL and aβ2GPI utilized specific reagents for each antibody (Phadia AB, Uppsala, Sweden) on the Phadia 250 (ThermoFisher Scientific, Massachusetts, Sweden).

Positive results were confirmed in accordance with the manufacturer’s interpretation manual. Furthermore, aPLA confirmations followed the guidelines from the International Society of Thrombosis and Hemostasis.^[12]^ LA detection on 2 or more occasions, spaced at least 12 weeks apart, signifies the presence of LA. Additionally, cases with aCL (IgG or IgM) levels exceeding 40 IgG or IgM phospholipid units, and aβ2GPI (IgG or IgM) titers surpassing the 99th percentile are classified as “medium-high titers.” Consequently, a high-risk aPLA profile is articulated as the presence of LA, or double positivity (any combination of LA, aCL antibody, or aβ2GPI), or triple aPLA positivity, or persistently high aPLA titers. Profiles of aCL or aβ2GPI alone at low and intermediate titers, particularly those transiently positive, are regarded as low-risk.^[13]^

The risk of thrombosis in aPLA-positive patients was computed using the adjusted global antiphospholipid syndrome score (aGAPSS), a tool for assessing risk. The aGAPSS assign points based on specific risk factors as follows: hyperlipidemia accounts for 3 points, arterial hypertension for 1 point, aCL IgG/IgM positivity for 5 points, aβ2GPI IgG/IgM positivity for 4 points, and LA positivity for 4 points.^[14]^

## 4. Aggravation of vessel stenosis

**Figure 2.**
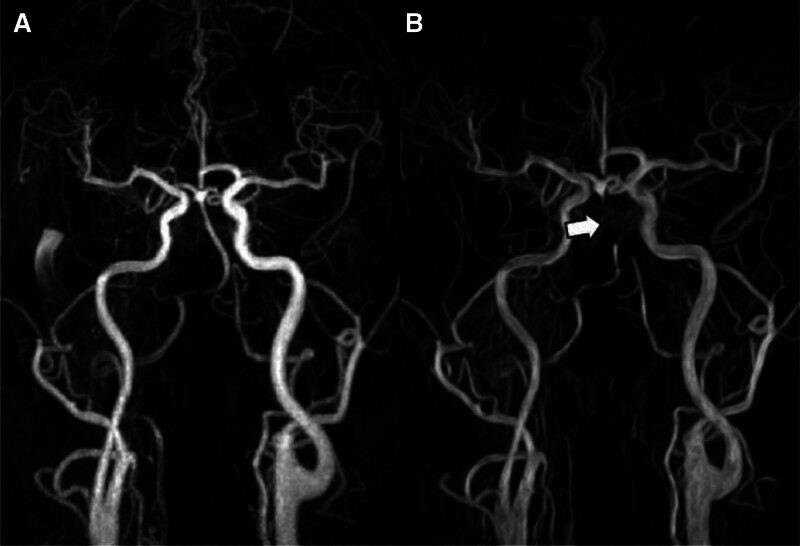
Cases in which cerebrovascular lesions progressed during the follow-up period were classified as “aggravation” as follows. For example, in a 72-year-old male patient, progression of basilar artery lesions was observed on follow-up magnetic resonance angiography 2 years and 2 months later (white arrow) in both (A) and (B).

Among the 647 patients confirmed to be positive for aPLA during this study period, the imaging tests of 202 patients with confirmed cerebral infarction were retrospectively reviewed. Each case was verified by at least 2 coauthors from the Department of Neurology or Neurosurgery who participated in this study. Aggravation was defined as the progression of vascular stenosis observed in the follow-up examination compared to the initial brain MRA performed at diagnosis, as illustrated in Figure 2.

## 5. Statistical analysis

We utilized SPSS version 25.0 (IBM Corp., Armonk, NY, USA) to compare continuous variables between 2 groups by employing the Student *t*-test or the Mann–Whitney *U* test, as appropriate. Categorical variables were analyzed using Fishers exact test or the Chi-square test. To mitigate the impact of baseline characteristic imbalances that could bias the outcomes due to risk factors associated with cerebral infarction other than aPLA testing, PSM was implemented. PSM was individually executed for each patient utilizing the nearest neighbor package in R version 4.2.2 (R Foundation for Statistical Computing, Vienna, Austria), considering variables such as age, sex, hypertension, and smoking habits. Values of *P* < .05 were considered statistically significant, and both ORs and 95% CIs were also reported.

## 6. Results

### 6.1. Baseline characteristics and risk factors of aPLA-positive patients: using PSM

The study compiled data on 647 aPLA-positive patients. The participants’ mean age was 65.8 ± 15.2 years, spanning from 15 to 98 years, with about 60% (393/647) being male. A predominant 95.2% of cases tested positive for LA, whereas aCL and aβ2GPI were positive in 8.8% and 5.3% of the cases, respectively. Additionally, high-risk and low-risk profiles of aPLA were identified in 7.1% and 8.3% of the cases, respectively. Table 1 summarizes the patients medical histories including positive aPLA factors. The analysis stratified patients into 2 distinct groups: 445 patients without cerebral infarction (mean age, 64.1 ± 15.8 years) and 202 patients with cerebral infarction (average age, 69.5 ± 13.1 years). In the cerebral infarction cohort, factors such as older age, male gender, high blood pressure, and smoking were significantly more prevalent. A matched subgroup was formulated for each of the 202 cases using 1:1 PSM to adjust for these differential risk factors, prompting further comparative analysis. Subsequent findings revealed that the history of smoking, aCL positivity, medium-high levels of aCL/aβ2GPI, or the presence of LA, along with both high and low-risk aPLA profiles, were significantly higher in the cerebral infarction group.

**Table 1:** Demographic and clinical characteristics before and after propensity score matching (PSM) based on the presence or absence of cerebral infarction in aPLA-positive patients.

	Positive aPLA				Propensity score matching (n = 404)		
	Overall (n = 647)	Without infarction (n = 445)	With infarction (n = 202)	*P* value	Without infarction (n = 202)	With infarction (n = 202)	*P* value
Age (yr)	*65.8 ± 15.2*	*64.1 ± 15.8*	*69.5 ± 13.1*	*.000*	69.1 ± 15.0	69.5 ± 13.1	.810
Male	*393 (60.7%*)	*257 (57.8%*)	*136 (67.3%*)	*.021*	129 (63.9%)	136 (67.3%)	.464
HTN	*370 (57.2%*)	*227 (51.0%*)	*143 (70.8%*)	*.000*	147 (72.8%)	143 (70.8%)	.658
DM	134 (20.7%)	86 (19.3%)	48 (23.8%)	.197	49 (24.3%)	48 (23.8%)	.907
Dyslipidemia	137 (21.2%)	94 (21.1%)	43 (21.3%)	.962	38 (18.8%)	43 (21.3%)	.534
Smoking	*178 (27.5%*)	*96 (21.6%*)	*82 (40.6%*)	*.000*	*63 (31.2%*)	*82 (40.6%*)	*.049*
Heart disease	158 (24.4%)	107 (24.0%)	51 (25.2%)	.741	49 (24.3%)	51 (25.2%)	.818
aPLA							
Positive LA	616 (95.2%)	427 (96.0%)	189 (93.6%)	.187	194 (96.0%)	189 (93.6%)	.262
Presence of LA	31 (4.8%)	16 (3.6%)	15 (7.4%)	.035	8 (4.0%)	15 (7.4%)	.133
Positive aCL	*57 (8.8%*)	*30 (6.7%*)	*27 (13.4%*)	*.006*	*10 (5.0%*)	*27 (13.4%*)	*.003*
Medium-high aCL	8 (1.2%)	4 (0.9%)	4 (2.0%)	.249	1 (0.5%)	4 (2.0%)	.177
Positive aβ_2_GPI	34 (5.3%)	20 (4.5%)	14 (6.9%)	.198	11 (5.4%)	14 (6.9%)	.536
Medium-high aβ_2_GPI	26 (4.0%)	15 (3.4%)	11 (5.4%)	.213	7 (3.5%)	11 (5.4%)	.335
High-risk aPLA profile	*46 (7.1%*)	*25 (5.6%*)	*21 (10.4%*)	*.028*	*10 (5.0%*)	*21 (10.4%*)	*.040*
Low-risk aPLA profile	*54 (8.3%*)	*29 (6.5%*)	*25 (12.4%*)	*.013*	*12 (5.9%*)	*25 (12.4%*)	*.025*
aGAPSS	5.2 ± 1.9	5.2 ± 1.8	5.4 ± 2.0	.124	5.3 ± 1.7	5.4 ± 2.0	.560

Values with *P* < 0.05 are italicized.

aCL = anticardiolipin antibodies, aGAPSS = adjusted global antiphospholipid syndrome score, aPLA = antiphospholipid antibodies, aβ_2_GPI = antiβ2-glycoprotein 1 antibodies, DM = Diabetes mellitus, HTN = Hypertension, LA = Lupus anticoagulant.

## 7. Risk factors for cerebral infarction in aPLA-positive patients: using logistic regression analyses

**Figure 3.**
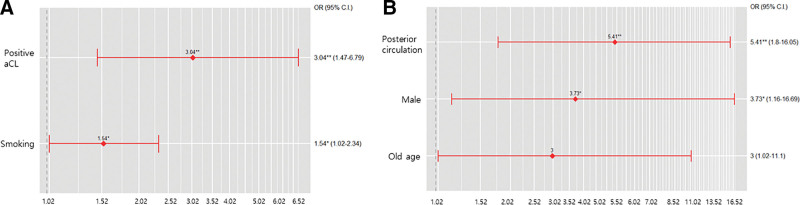
Logistic regression plot of odds ratios and 95% CIs. (A) multivariate logistic regression analysis of risk factors associated with infarction in aPLA- positive patients; (B) multivariate logistic regression analysis of risk factors associated with the aggravation of vessel stenosis in aPLA-positive patients with cerebral infarction.

To identify risk factors for cerebral infarction among patients with the aPLA positivity, we employed both univariate and multivariate logistic regression analyses on subjects post-PSM. As indicated in Table 2, no significant correlation was observed with other factors. However, smoking history and aCL positivity were associated with notably increased risks, being 1.5 and 3 times higher, respectively (OR = 1.543; 95% CI: 1.020–2.334; *P* = .040, OR = 3.043; 95% CI: 1.426–6.491; *P* = .040, respectively) (Fig. 3).

**Table 2:** Univariate and multivariate logistic regression analysis of risk factors associated with infarction in aPLA-positive patients after propensity score matching.

	Univariate analysis		Multivariate analysis	
	OR (95% CI)	*P* value	OR (95% CI)	*P* value
Old aged (≥65)	1.099 (0.718, 1.680)	.665		
Male	1.166 (0.773, 1.759)	.464		
HTN	0.907 (0.588, 1.399)	.658		
DM	0.973 (0.616, 1.536)	.907		
Dyslipidemia	1.167 (0.717, 1.901)	.535		
Smoking	*1.508 (1.001, 2.270*)	*.049*	*1.543 (1.020, 2.334*)	*.040*
Heart disease	1.055 (0.671, 1.657)	.818		
aPLA				
Positive LA	0.600 (0.243, 1.479)	.267		
Presence of LA	1.945 (0.806, 4.696)	.139		
Positive aCL	*2.962 (1.394, 6.296*)	*.005*	*3.043 (1.426, 6.491*)	*.040*
Medium-high aCL	4.061 (0.450, 36.650)	.212		
Positive aβ_2_GPI	1.293 (0.572, 2.921)	.537		
Medium-high aβ_2_GPI	1.604 (0.609, 4.225)	.339		
High-risk aPLA profile	*2.228 (1.021, 4.859*)	*.044*		
Low-risk aPLA profile	*2.236 (1.091, 4.586*)	*.028*		
Posterior circulation	1.130 (0.569, 2.243)	.727		

Values with *P* < 0.05 are italicized.

aCL = anticardiolipin antibodies, aGAPSS = adjusted global antiphospholipid syndrome score, aPLA = antiphospholipid antibodies, aβ_2_GPI = antiβ2-glycoprotein 1 antibodies, DM = Diabetes mellitus, FU = follow-up, HTN = Hypertension, LA = Lupus anticoagulant.

## 8. Univariate and multivariate logistic regression analyses for vascular aggravation

During a follow-up period of 16.3 ± 24.1 months (range 0–114 months) among 202 cerebral infarction cases, a new subgroup comprising 25 patients with worsening vascular stenosis was identified, as shown in Figure 2, and a subanalysis was conducted on this subgroup. As detailed in Table 3, both univariate and multivariate logistic regression analyses were performed to determine risk factors for vessel aggravation. The multivariate analysis displayed a nonsignificant trend toward a higher prevalence among patients over 65 years of age. Moreover, being male was identified as being associated with a 3.7 times higher risk (OR = 3.73; 95% CI: 1.16–16.69; *P* = .046), and involvement of the posterior circulation was associated with a 5.4 times higher risk than anterior circulation involvement (OR = 5.41; 95% CI: 1.80–16.05; *P* = .002) (Fig. 3).

**Table 3:** Univariate and multivariate logistic regression analysis of risk factors associated with the aggravation of vessel stenosis in aPLA-positive patients with cerebral infarction.

	Aggravation (%)		Univariate analysis		Multivariate analysis	
	No (n = 177)	Yes (n = 25)	OR (95% CI)	*P* value	OR (95% CI)	*P* value
Age (yr)	69.2 ± 13.3	71.6 ± 11.5	1.015 (0.982–1.051)	.389		
Old aged (≥65)	122 (68.9%)	21 (84.0%)	2.37 (0.776–7.222)	.130	3.00 (1.02–11.10)	.065
Male	114 (64.4%)	22 (88.0%)	*4.05 (1.167–14.073*)	.*028*	**3.73 (1.16–16.69**)	**.046**
HTN	127 (71.8%)	16 (64.0%)	0.700 (0.290–1.687)	.427		
DM	39 (22.0%)	9 (36.0%)	1.990 (0.817–4.850)	.130		
Dyslipidemia	37 (20.9%)	6 (24.0%)	1.195 (0.445–3.205)	.724		
Smoking	70 (39.5%)	12 (48.0%)	1.411 (0.609–3.270)	.422		
Heart disease	46 (26.0%)	5 (20.0%)	0.712 (0.253–2.006)	.520		
aPLA						
Presence of LA	14 (7.9%)	1 (4.0%)	0.495 (0.061–3.858)	.494		
Medium-high aCL	4 (2.3%)	0 (0.0%)	0.000	.999		
Medium-high aβ_2_GPI	9 (5.1%)	2 (8.0%)	1.623 (0.330–7.993)	.551		
High-risk aPLA profile	19 (10.7%)	2 (8.0%)	0.723 (0.158–3.310)	.676		
Low-risk aPLA profile	23 (13.0%)	2 (8.0%)	0.582 (0.129–2.635)	.483		
aGAPSS	5.4 ± 2.0	5.7 ± 2.1	1.074 (0.885–1.303)	.471		
Location						
Anterior circulation	166 (93.8%)	17 (68.0%)				
Posterior circulation	11 (6.2%)	8 (32.0%)	*7.102 (2.514–20.062*)	*.000*	*5.41 (1.80–16.05*)	*.002*
FU duration(months)	19.4 ± 24.4	35.4 ± 27.8	1.022 (1.007–1.037)	.005		

Values with *P* < 0.05 are italicized.

aCL = anticardiolipin antibodies, aGAPSS = adjusted global antiphospholipid syndrome score, aPLA = antiphospholipid antibodies, aβ_2_GPI = antiβ2-glycoprotein 1 antibodies, DM = Diabetes mellitus, FU = follow-up, HTN = Hypertension, LA = Lupus anticoagulant.

## 9. Discussion

In the TOAST classification of cerebral infarction, APS have traditionally been categorized as “unknown negative” or “other determined.” However, given the diverse etiologies associated with APS and the mounting evidence linking aPLA to cerebral infarction, it is more accurate to classify APS-related strokes under large artery atherosclerosis and small vessel occlusion.^[4,7,15,16]^ Consequently, our study posits that it is more appropriate to consider the presence of aPLA as an independent risk factor for stroke, irrespective of other established risk factors. This assumption supports the use of PSM analysis in conjunction with a clinical approach to further refine and pinpoint additional risk factors for cerebral infarction. This study is pioneering in identifying statistically significant factors that contribute to vascular stenosis progression in patients positive for aPLA.

We found that the predominant aPLA factor associated with cerebral infarction is was revealed to be aCL. The notable correlation of both high-risk and low-risk aPLA profiles with cerebral infarction likely arises from their inclusion of aCL positivity. Nevertheless, the specific mechanisms through which aCL is synthesized and its exact modes of action remain poorly understood. One theory suggests that endothelial cell damage, induced by other stroke risk factors like hypertension, may reveal antigens concealed within the phospholipid bilayer of the cell membrane, thereby eliciting an immune response involving aCL, which has affinity for endothelial cells.^[17,18]^

Further studies have demonstrated its effects on altered coagulation tests, including prolonged activated partial thromboplastin times, reduced antithrombin III (AT III) activity, and increased levels of D-Dimer and fibrinogen degradation products in aCL-positive cerebral infarction patients. These changes reflect inhibited activated protein C activity and diminished inactivation of factors V and VIII. Additionally, aCL might hinder fibrinolysis by suppressing AT III activity and Kallikrein activation, thus fostering thrombosis.^[19–21]^ Nonetheless, the relevance of detecting aCL in stroke patients is underscored by the retrospective nature of our study, which primarily conducted aCL testing postthrombosis, suggesting aCL may develop subsequent to vascular injury from the stroke.^[22,23]^

Our study’s findings on the association between smoking and stroke in aPLA patients align with previous research; studies indicate that smoking rates among APS patients vary 30% to 50%.^[24,25]^ Our analysis also revealed that approximately 40% of aPLA-positive stroke patients had a history of smoking. According to Fickl et al (1996), smoking may exacerbate atherosclerosis progression by promoting autoantibody formation against cardiolipin and ox-LDL. Since then, numerous studies have demonstrated that smoking is associated with cardiovascular disease and is an important predictor thereof.^[26–28]^ The precise mechanism through which smoking may induce aPLA production remains unclear; however, 1 hypothesis suggests that chronic exposure to cigarettes results in endothelial damage, which in turn stimulates aPLA responses through exposure to antigens hidden within the phospholipid bilayer. Keswanie and Chauhan have supported this hypothesis, noting that young patients presenting with stroke or transient ischemic attack who consistently exhibited high aCL IgG levels were predominantly smokers.^[29–31]^ Additionally, results from our study’s logistic regression analysis confirmed that smoking and aCL positivity are major risk factors for cerebral infarction in patients.

To the best of our knowledge, our study is the first to identify significant risk factors for the progression of aPLA-related cerebrovascular stenosis. A 2022 study by Yeo et al, involving 11 participants, indicated that proliferative vasculopathy (PV) – aPLA does not show a preference for women, as opposed to classic APS-related stroke, which is 3 times more prevalent in women.^[32,33]^ Conversely, our study found that men experienced nearly 4 times as many vascular aggravations, establishing gender as a significant risk factor. Additionally, our findings identified the posterior circulation as a decisive factor, though previous studies have not explored vessel location and vascular lesion progression to this extent. Angiographic findings from several other studies have illustrated extensive long-segment stenosis in medium-sized extracranial and intracranial arteries, such as the carotid, basilar, and proximal cerebral arteries, alongside multiple short-segment, abrupt stenosis and distal occlusions. These associations are typically concurrent with atheromatous or thrombotic lesions.^[34,35]^ Currently, there are no formal management guidelines for PV-aPLA. However, recommendations from the 13th International Conference on Antiphospholipid Antibodies suggest that patients with APS and arterial thrombosis should receive warfarin to maintain a PT-INR > 3.0, or a combination of low-dose aspirin and standard-strength warfarin (PT-INR range, 2.0–3.0). Treatment should be considered accordingly, but additional research is needed to define the optimal treatment for PV-aPLA.^[33,36,37]^

This study has several limitations. Firstly, the data were collected and analyzed retrospectively from a single institution, and although statistical adjustment was attempted using PSM, the results may still be subject to bias. Secondly, the recruitment of patients who underwent aPLA testing was heterogeneous, including those with headaches and suspected TIA or stroke. The group of patients with cerebral infarction could also be further categorized by various causes, but in this study, it was simply classified as presence or absence, lacking a more detailed and accurate analysis of factors according to each etiology. Lastly, the definition of cerebrovascular stenosis, a key outcome of this study, was established through discussions between 2 or more authors specializing in neurology and neurosurgery, which may not fully guarantee objectivity and freedom from errors. To enhance the objectivity and accuracy of this study, long-term follow-up results and prospective multicenter randomized controlled trials are required.

## 10. Conclusion

In this study, factors related to cerebral infarction in aPLA-positive patients were analyzed, adjusting for variables such as age, gender, hypertension, and smoking. Both smoking and aCL positivity emerged as principal factors associated with cerebral infarction. Furthermore, logistic regression and subgroup analysis on factors exacerbating cerebrovascular stenosis revealed that male gender and posterior circulation are significant risk contributors to vascular aggravation. Nonetheless, further well-designed prospective studies focusing on aPLA-related cerebrovascular disease are imperative.

## Author contributions

**Conceptualization:** Seung Hyun Ha.

**Data curation:** Sang-Uk Kim, Joon Huh.

**Methodology:** Sang-Uk Kim.

**Resources:** Choon-Woong Huh.

**Supervision:** Choon-Woong Huh.

**Writing – original draft:** Seung Hyun Ha.

**Writing – review & editing:** Sang-Uk Kim, Joon Huh, Choon-Woong Huh.
